# Application of Oblique Lateral Interbody Fusion Combined with Bridge‐Locking Cage in Adjacent Segment Disease After Lumbar Fusion

**DOI:** 10.1111/os.13449

**Published:** 2022-10-21

**Authors:** Shuai Zhang, Hui Xu, Cheng‐hui Yin

**Affiliations:** ^1^ Department of Orthopedics 900th Hospital of PLA Fuzhou China

**Keywords:** Adjacent segment disease, Bridge‐locking cage, Lumbar fusion, Oblique lateral interbody fusion, Therapeutic efficacy

## Abstract

**Objective:**

Adjacent segment disease (ASD) is considered any abnormal process that develops in the mobile segment next to spinal fusion, accompanied by related symptoms. To evaluate the efficacy and complications of oblique lateral interbody fusion (OLIF) combined with bridge‐locking fusion on ASD after lumbar fusion.

**Methods:**

A total of 35 ASD patients who required re‐operation after lumbar fusion in our hospital from March 2014 to March 2020 were retrospectively analyzed, among which 13 cases (seven males and six females; 62.3 ± 11.3 years old) received the treatment of OLIF + bridge‐locking cage internal fixation (OLIF group), and 22 cases (14 males and eight females; 52.3 ± 17.8 years old) received the treatment of transforaminal lumbar interbody fusion (TLIF) + pedicle screw fixation (TLIF group). The comparison of the operation time, intraoperative blood loss, postoperative drainage volume, and length of hospital stay between the two groups of patients was performed using the *t*‐test. The comparison of Visual Analogue Scale (VAS) and Oswestry Disability Index (ODI) at different time points before and after the operation was performed using analysis of variance for repeated measurement data. The fusion rate and postoperative complications of the two groups of patients were also evaluated.

**Results:**

The operation time of patients in the OLIF group (55.4 ± 12.4 min) was significantly shorter than that of patients in the TLIF group (94.3 ± 22.9 min) (*P* < 0.05), the length of stay of patients in the OLIF group (7.4 ± 2.3 day) was significantly shorter than that of patients in the TLIF group (12.4 ± 3.2 day) (*P* < 0.05); the intraoperative blood loss (62.2 ± 30.1 mL) and postoperative drainage (47.3 ± 22.4 mL) of patients in the OLIF group were significantly less than those of patients in the TLIF group with intraoperative blood loss (363.4 ± 120.2 mL) and postoperative drainage (285.5 ± 57.8 mL) (all *P*s < 0.05). Besides, the VAS and ODI scores of the two groups of patients were improved 3 months after the operation and at the last follow‐up (all *P*s < 0.05). Three patients in the OLIF group developed complications such as hip flexion weakness and fusion cage sink, with an incidence of 23.1%. Three patients in the TLIF group developed complications including wound infection and intraoperative nerve injury, with an incidence of 22.7%.

**Conclusion:**

The combination of OLIF and bridge‐locking cage may be a safe and effective therapy for ASD patients after lumbar fusion operation.

## Graphical abstract

Sketch map of ROI‐A fusion cage. The self‐locking double fixation inlay designed on special PEEK fusion cage (A) was directly implanted on the axis of intervertebral space (B), firmly fixed to the upper and lower vertebral bodies through the vertebral endplate, and then locked on the fusion cage (C).

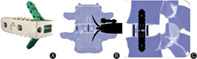

## Introduction

Adjacent segment disease (ASD) is defined as a degenerative disorder occurring in the cranial or caudal vertebral body at the lumbar fusion segment, which constitutes one of the most salient complications after posterior lumbar fusion.^1^ ASD is considered any abnormal process that develops in the mobile segment next to spinal fusion, accompanied by related symptoms, such as radiculopathy, myelopathy, instability and so on.^2^, ^3^ Changed biomechanics near the previous fusion site predispose to degenerative changes, and the existing spondylosis at adjacent levels are associated with the occurrence of ASD.^4^, ^5^, ^6^, ^7^ Clinically, ASD is primarily manifested as unilateral disc herniation, which leads to lower extremity nerve root pain and seriously affects the quality of life of patients. Thus, it makes ASD a vital contributor to reoperation after lumbar fusion surgery.^8^ Posterior lumbar fusion is accepted as a common method for the treatment of lumbar degenerative diseases. Posterolateral lumbar fusion (PLF) and posterior lumbar interbody fusion (PLIF) are the most widely used open posterior lumbar interbody fusion in the clinic.

In recent years, percutaneous transforaminal endoscopic discectomy is extensively used for the treatment of ASD.^9^ However, if ASD patients present spinal stenosis and lumbar instability, it is necessary to perform fusion again in the diseased segments. Although numerous studies have shown that the traditional lumbar fusion operations including PLF, PLIF, transforaminal lumbar interbody fusion (TLIF) and anterior lumbar interbody fusion (ALIF) can provide favorable clinical outcomes and radiographic alignment for ASD patients with L5/S1 segment after lumbar fusion, some studies reported some disadvantages about these traditional operations. For instance, PLF's effect on discogenic low back pain is still controversial; PLIF may have a role to stretch the dural sac and nerve root; TLIF may cause damage to the nerve root, and with incomplete discectomy; ALIF has a relatively higher risk, including the injury of large blood vessels and visceral nerves during operation, also, ALIF may lead to abdominal distension, reverse ejaculation and urination dysfunction after operation.^10^, ^11^ Several reasons make it more difficult for ASD patients to undergo open surgery again. For example, scar formation in the operation area leads to unclear anatomical structure; adhesion of nerve root and dura mater leads to incomplete decompression and increased bleeding; operation again causes great trauma and requires a long recovery time.^12^, ^13^, ^14^

Oblique lateral interbody fusion (OLIF) emerges as a minimally invasive operation in recent years for various lumbar spinal degenerative diseases, such as lumbar degenerative scoliosis, mild‐to‐moderate spinal stenosis, spondylolisthesis, and lumbar instability.^15^ OLIF was introduced by Silvestre *et al*. in 2012, which evolved from direct (extreme) lateral transposes interbody fusion (DLIF or XLIF) and ALIF.^16^ OLIF accesses the lumbar spine through the anatomical space between the aorta (or inferior vena cava) and the psoas to avoid causing damages on lumbar plexus nerve and blood vessels.^15^ Thus, unlike the traditional surgery, OLIF is less likely to cause damages to the lamina, para spinal muscles, facet joints, and lumbar plexus. It has been reported that OLIF can produce excellent clinical results with a high fusion rate and a low incidence rate of complications compared with other traditional procedures like LLIF and ALIF.^17^ Several studies have reported that OLIF possesses the advantages of short operation time, less blood loss, effective deformity correction, rapid postoperative recovery, and good clinical effects.^18^, ^19^, ^20^ The utility of OLIF for correcting deformity has also been reported.^21^ However, several studies have reported that there are some complications associated with OLIF. According to the systematic review performed by Li *et al*., among 1453 patients who underwent OLIF, vascular injury is the most common intraoperative complication.^22^ The symptom of lower extremity incision pain caused by sympathetic chain injury after OLIF is also reported.^23^

Bridge‐locking cage (Avenue‐L, Zimmer Biomet, Warsaw, IN, USA) is a self‐locking double fixation inlay designed on the special PEEK fusion device, with zero profile, which can be directly implanted on the axis of intervertebral space, firmly fixed to the upper and lower vertebral bodies through the vertebral endplate, and then locked on the fusion cage, thus obtaining immediate stability.

Currently, the clinical application of OLIF focuses more on primary lumbar degenerative diseases than ASD.^24^, ^25^ Against this backdrop, this study followed up and analyzed the ASD patients undergoing operation again after lumbar fusion. The research objective of this study is as follow: first, to compare the clinical efficacy of OLIF + bridge‐locking cage internal fixation and TLIF + pedicle screw fixation on the treatment of ASD, secondly, to investigate the advantages and complications of OLIF + bridge‐locking cage internal fixation in the treatment of ASD, and third, to provide a theoretical basis for the treatment of ASD after lumbar fusion.

## Methods

### General Information

A total of 35 ASD patients who required re‐operation after lumbar fusion in our hospital from March 2014 to March 2020 were retrospectively analyzed. These patients received OLIF + bridge‐locking cage internal fixation (OLIF group) or TLIF + pedicle screw fixation (TLIF group), including 13 cases (seven males and six females; 62.3 ± 11.3 years old) in the OLIF group and 22 cases (14 males and eight females; 2.3 ± 17.8 years old) in the TLIF group. All patients were operated by the same surgeon. The inclusion criteria were as follows:^26^ (i) ASD patients after receiving lumbar fusion surgery; (ii) patients planning to under gore‐operation of OLIF + bridge‐locking cage internal fixation or TLIF + pedicle screw fixation; (iii) patients with complete imaging data and follow‐up data;(iv) patients clinically presented intermittent claudication or low back pain accompanied by lower extremity nerve root pain, and these symptoms were relieved after bed rest; imaging findings showed single segment spinal stenosis, lumbar instability, and disc herniation caused by adjacent segment degeneration after lumbar fusion operation; and (v) more than 3 months of systematic conservative treatment did not produce good curative effects, and the patient's life and work were seriously affected. The exclusion criteria were as follows: (i) patients with traumatic, pathological, or iatrogenic spondylolisthesis, and patients who had received lumbar spine surgery before; (ii) patients with concomitant scoliosis; (iii) vertebral fracture or instability at the adjacent segments; and (iv) herniated lumbar disc or lumbar spondylolisthesis ≥2 degrees. This study was approved by the medical ethics committee of the 900th Hospital of the Joint Logistic Support Force (2021 medical ethics review 012).

### Operation Methods

#### OLIF Group

**Fig. 1:**
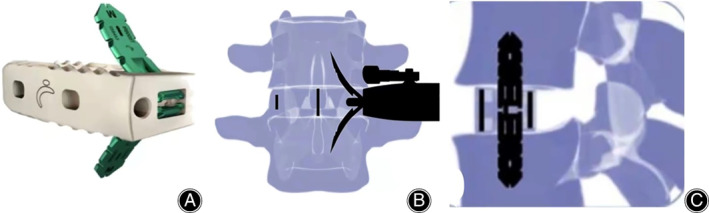
Sketch map of ROI‐A fusion cage. The self‐locking double fixation inlay designed on special PEEK fusion cage (A) is directly implanted on the axis of intervertebral space (B), firmly fixed to the upper and lower vertebral bodies through the vertebral endplate, and then locked on the fusion cage (C)

**Fig. 2:**
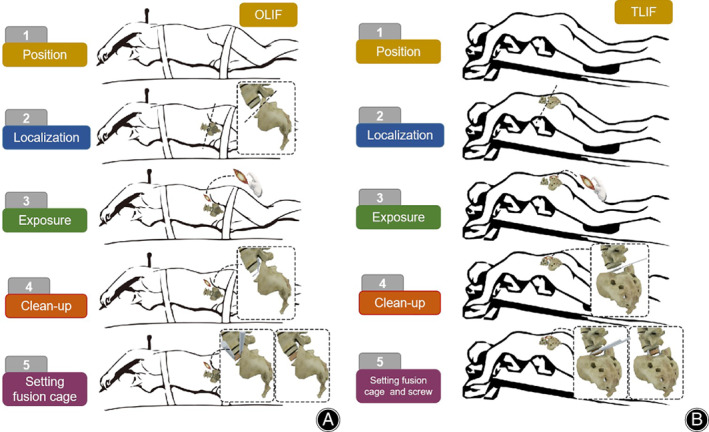
(A) Schematic of OLIF procedure. (B) Schematic of TLIF procedure

The patients were in a right recumbent position with the body fixed firmly, followed by routine disinfection and draping. Briefly, (i) a 10 cm incision was made on the left anterior side of the lumbar spine (target segment). The skin and subcutaneous tissue were cut and electrocoagulation was used for hemostasis. The external oblique muscle, internal oblique muscle, and transverse muscle of the abdomen were incised layer by layer to fully expose the target space. (ii) The positioning needle was placed in the target segment space, and the C‐arm fluoroscopy confirmed that the positioning needle was located in the target segment space. (iii) The annulus fibrosus was cut with a small round knife, and the nucleus was removed with nucleus forceps. (iv) The intervertebral disc tissues and the upper and lower endplates were fully scraped off with a long handle spatula until the subchondral bone was exposed for complete decompression and bone bed preparation. (v) The allogeneic bone was packed into the fusion cage, and the intervertebral fusion cage (Avenue‐L) with appropriate height was inserted into the target intervertebral space. (vi) After the C‐arm fluoroscopy confirmed the proper location of the fusion cage, a fixation inlay was inserted upward and downward respectively. Again, the C‐arm fluoroscopy confirmed the proper location of fixation inlay. (vii) The incision was rinsed repeatedly and a drainage tube was placed. (viii) Finally, the transverse muscle, internal oblique muscle, and external oblique muscle of the abdomen, subcutaneous tissues, and skin were sutured. The schematic of OLIF surgical procedure is shown in Figs 1 and 2A.

#### TLIF Group

The patients were in a prone position, followed by routine disinfection and draping. The detailed procedure was follows: (i) two longitudinal incisions (4–6 cm) were made along the midline of the target segment. The skin and subcutaneous and deep fascia were cut, and the muscular space between the longissimus muscle and multifidus muscle was blunt separated. (ii) The articular process was exposed and the entry point of pedicle screws was determined. A sharp cone was used to make an opening, and an opening device was used to explore the screw path through the pedicle. (iii) Then, pedicle screws with appropriate diameter and length were placed. The C‐arm fluoroscopy confirmed the internal fixation position. (iv) According to the preoperative diagnosis, the inferior articular process and part of the superior articular process were resected with an osteotome and laminectomy punch for adequate for aminal decompression. The lower part of the lamina and the dorsal ligamentum flavum of canalis vertebralis were removed for adequate for aminal decompression. (v) The intervertebral disc was exposed. The annulus fibrosus was cut with a small round knife, and the nucleus was removed with nucleus forceps. The intervertebral disc tissues and the upper and lower endplates were fully scraped off with a long handle spatula until the subchondral bone was exposed, and the bone bed was prepared. (vi) The interbody fusion cage with suitable height was placed and the rods were re‐implanted connecting L3‐L5 screws. Again, the C‐arm fluoroscopy confirmed the proper location of the fusion cage. (vii) The incision was rinsed repeatedly and a drainage tube was placed. (viii) Finally, the incision was sutured. The schematic of TLIF surgical procedure is shown in Fig. 2B.

The drainage tube was removed on the first day after the operation. On the second day after the operation, the patients began to walk and move properly under the protection of lumbar support. The patients were required to wear lumbar support within 2 months after the operation and avoid severe lumbar twisting and bending activities.

### Clinical Efficacy Evaluation

The related‐indexes during the preoperative, perioperative, and postoperative period were recorded. (i) Indexes recorded during the preoperative period: visual analogue scale (VAS) and Oswestry disability index (ODI); (ii) indexes recorded during the perioperative period: operation time, intraoperative blood loss, postoperative drainage volume, and hospital stay; (iii) indexes recorded during the postoperative period: VAS, ODI, complications, further treatments, remission, and recovery. The early complications such as intraoperative nerve injury, postoperative incision infection, postoperative cerebrospinal fluid leakage, postoperative hip flexion weakness, and lower limb numbness or pain were observed. The late complications such as fusion cage displacement and sink and breakage of internal fixation were detected. VAS is one of the most commonly used index to measure the degree of pain^27^ and ODI is a condition‐specific outcome measure for spinal disorders.^28^

### Imaging Efficacy Evaluation

On the second day after the operation, all patients received MR examination of frontal and lateral of lumbar vertebra to confirm the position of the fusion cage. All patients were followed up 3, 6, and 12 months after the operation. The frontal and lateral X‐ray films and lumbar CT were reviewed to evaluate the stability of the cage and the success rate of bone graft fusion.

### Statistical Analysis

Statistical analysis was performed by using SPSS 25.0 (IBM Corp., Armonk, NY, USA). The measurement data were in normal distribution and expressed as mean ± standard deviation. The comparison of the operation time, intraoperative blood loss, postoperative drainage volume, and length of hospital stay between the two groups of patients was performed using the *t*‐test. The comparison of VAS and ODI at different time points before and after the operation was performed using analysis of variance for repeated measurement data. The *P*‐value was obtained from a two‐tailed test, and *P* < 0.05 meant statistical significance.

## Results

### Comparison of the Operation Time, Intraoperative Blood Loss, Postoperative Drainage, and Hospital Stay

The operation was successfully performed in the two groups of patients. The symptoms such as postoperative low back pain and lower limb pain numbness were notably alleviated. The operation time of patients in the OLIF group (55.4 ± 12.4 min) was significantly shorter than that of patients in the TLIF group (94.3 ± 22.9 min) (*t* = 12.24, *P* < 0.05); the intraoperative blood loss (62.2 ± 30.1 mL) and postoperative drainage (47.3 ± 22.4 mL) of patients in the OLIF group were significantly less than those of patients in the TLIF group with intraoperative blood loss (363.4 ± 120.2 mL) and postoperative drainage (285.5 ± 57.8 mL) (*t* = 25.66, *P* < 0.05); the length of stay of patients in the OLIF group (7.4 ± 2.3 day) was significantly shorter than that of patients in the TLIF group (12.4 ± 3.2 day) (*t* = 6.42, *P* < 0.05), as shown in Table 1.

**TABLE 1:** Comparison of perioperative related indexes between the two groups

Groups	Case	Operation time (min)	Intraoperative blood loss (mL)	Postoperative drainage volume (mL)	Hospital stay (days)
OLIF group	13	55.4 ± 12.4	62.2 ± 30.1	47.3 ± 22.4	7.4 ± 2.3
TLIF group	22	94.3 ± 22.9*	363.4 ± 120.2*	285.5 ± 57.8*	12.4 ± 3.2*
*t*		12.24	36.23	25.66	6.42
*p*		0.032	0.017	0.026	0.004

*Note*: *P < 0.05 *vs*. TLIF group.

### Comparison of VAS and ODI Scores

The VAS score of patients in the OLIF group was (7.8 ± 1.1) before the operation, and this figure decreased to (1.8 ± 0.2) 3 months after the operation and (0.65 ± 0.3) at the last follow‐up. The VAS score of patients in the TLIF group was (8.1 ± 1.0) before the operation, and this figure decreased to (1.9 ± 0.3) 3 months after the operation and (0.52 ± 0.2) at the last follow‐up. The VAS score of the two groups of patients at different time points was improved after operation compared with that before operation (*F* = 15.52, *P* < 0.05; *F* = 24.65, *P* < 0.05), but there was no significant difference between the two groups (*t* = 1.32, *p* = 1.24; *t* = 1.21, *P* = 0.98; *t* = 1.25, *P* = 1.12), as shown in Table 2.

**TABLE 2:** Comparison of VAS score between the two groups before and after operation

Groups	Case	Before operation	3 months after operation	The last follow‐up	*F*	*P*
OLIF group	13	7.8 ± 1.1	1.8 ± 0.2*	0.65 ± 0.3**	15.52	<0.05
TLIF group	22	8.1 ± 1.0	1.9 ± 0.3*	0.52 ± 0.2**	24.65	<0.05
*t*	—	1.32	1.21	1.25	—	—
*p*	—	1.24	0.98	1.12	—	—

Abbreviation: VAS, visual analogue scale.

*Note*: *p < 0.05 *vs*. before operation.

**p > 0.05 *vs*. TLIF group.

**Fig. 3:**
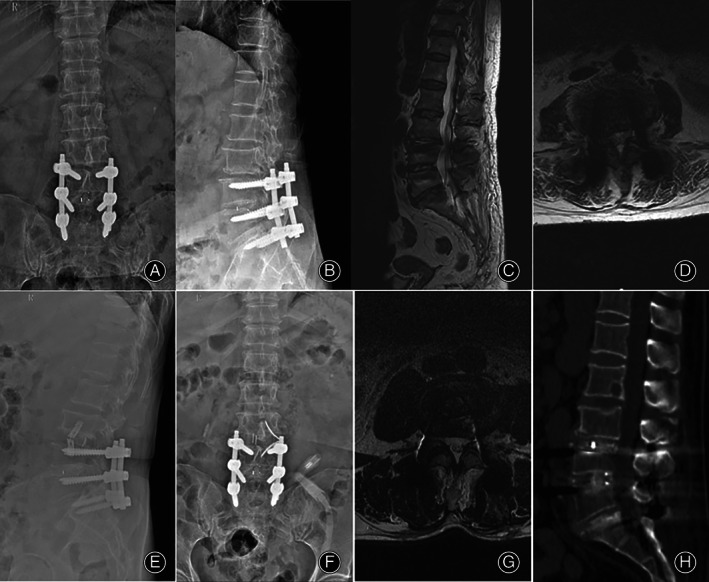
Imaging evaluation of the typical case in the OLIF group. A patient (female, 48 years old) was admitted to our hospital with the chief complaint of “6 years after lumbar surgery and intermittent claudication for 6 months.” After admission, she received OLIF + bridge‐locking cage internal fixation, and the postoperative symptoms were significantly relieved. (A–D) preoperative X‐ray and MRI indicate L3/4 spinal stenosis. (E–H) postoperative X‐ray, MRI, and CT show the increased area of L3/4 spinal canal and successful bone graft fusion

**Fig. 4:**
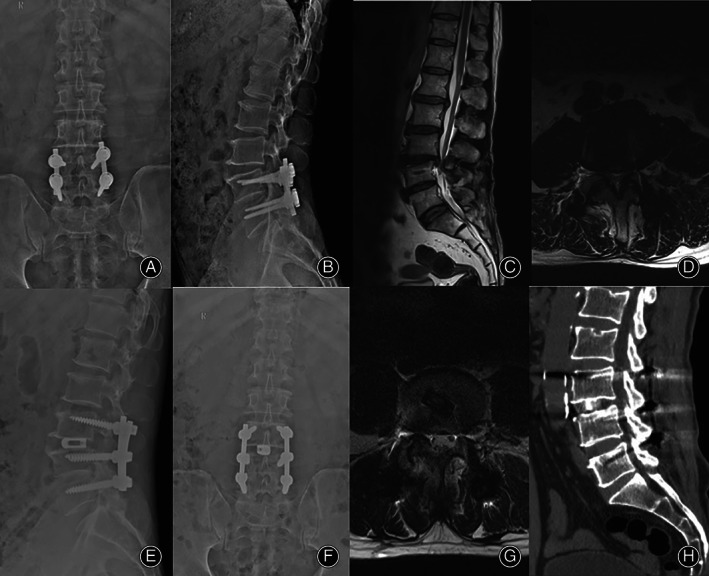
Imaging evaluation of the typical case in the TLIF group. A patient (male, 54 years old) was admitted to our hospital with the chief complaint of “10 years after lumbar surgery and numbness and pain of both lower limbs for 5 months.” After admission, he received TLIF + pedicle screw fixation, and the postoperative symptoms were significantly alleviated. (A–D) preoperative X‐ray and MRI indicate L3/4 spinal stenosis. (E–H) postoperative X‐ray, MRI, and CT show the increased area of L3/4 spinal canal and successful bone graft fusion

The ODI of patients in the OLIF group was 58.9% ± 10.8% before the operation, and this figure decreased to 9.6% ± 1.5% 3 months after the operation and 8.3 ± 0.7% at the last follow‐up. The ODI score of patients in the TLIF group was 57.4% ± 11.5% before the operation, and decreased to 10.9% ± 1.2% 3 months after the operation and 9.2 ± 0.8% at the last follow‐up (Figs 3A‐H and 4A‐H). The ODI score of the two groups of patients 3 months after the operation and at the last follow‐up was improved compared with that before the operation (*F* = 32.87, *P* < 0.05; *F* = 54.21, *P* < 0.05), but there was no significant difference between the two groups, as shown in Table 3.

**TABLE 3:** Comparison of ODI score between the two groups before and after operation

Groups	Case	Before operation	3 months after operation	The last follow‐up	*F*	*P*
OLIF group	13	58.9 ± 10.8	9.6 ± 1.5*	8.3 ± 0.7**	32.87	<0.05
TLIF group	22	57.4 ± 11.5	10.9 ± 1.2*	9.2 ± 0.8**	54.21	<0.05
*t*	—	0.89	1.04	0.98	—	—
*p*	—	0.56	1.05	0.75	—	—

Abbreviation: ODI, Oswestry Disability Index

*p < 0.05 *vs*. before operation.

**p > 0.05 *vs*. TLIF group.

### Comparison of Fusion Rate and Postoperative Complication

One year after the operation, the two groups of patients returned to the hospital for reexamination. It was found that all the fusion cages were fused in the upper and lower vertebral bodies with bone in growth, with a fusion rate of 100%. In the OLIF group, three patients developed postoperative complications, with an incidence rate of 23.1%.

Among them, one patient had hip flexion weakness after the operation, and the symptoms were relieved after intensive exercise and nutritional nerve treatment; two patients showed fusion cage sink, and the fusion cage did not continue to sink after external fixation and anti‐osteoporosis treatment. In the TLIF group, five patients developed postoperative complications, with an incidence rate of 22.7%. Among them, one patient had wound infection, and the wound healed well after anti‐infection and incision and drainage; four patients had intraoperative nerve injury, and three of them recovered within 3 months after the operation with the aid of intensive exercise and nutritional nerve treatment, but one of them did not fully recover at the last follow‐up.

## Discussion

Degenerative lumbar disease constitutes one of the common causes of low back and leg pain in the elderly. Posterior lumbar fusion is required in the presence of conservative treatment failure. ASD is the major complication of posterior lumbar fusion. If ASD is manifested as spinal stenosis and lumbar instability, it is necessary to re‐perform fusion operation in the diseased segment. TLIF is a commonly used lumbar fusion, while OLIF is an emerging minimally invasive operation in recent years. This study compared the operation time, intraoperative blood loss, postoperative drainage, hospital stay, VAS, and ODI of the patients who received TLIF or OLIF.

### Advantages and Disadvantages of OLIF

As a minimally invasive operation, OLIF + bridge‐locking cage internal fixation has the following advantages in the treatment of ASD. (i) The self‐locking double fixation inlay designed on the special PEEK fusion cage can enhance the stability of a simple OLIF fusion cage, thus achieving immediate stability, avoiding posterior internal fixation operation, and giving full play to the advantages of minimally invasive OLIF (stand alone). (ii) The fusion cage is made of PEEK material with the same elastic modulus as human bone. Combined with the initial stability provided by self‐locking inlay, it can effectively reduce the risk of sink and displacement of the fusion cage. (iii) During the implantation of inlay, the anterior column is slotted to provide blood for intervertebral fusion. Poor position of the fusion cage has become the most common complication after OLIF. In this study, 13 patients achieved bony fusion at the last follow‐up, without fusion cage displacement. However, two patients had fusion cage sink, with an incidence of 15.4%. One of them was an obese patient with the body mass index (BMI) ≥ 30 kg/m^2^, and the other had severe osteoporosis with the *t* value < −2.5. Attention must be paid to the evaluation of BMI and *t* value on the choice of operation. Previous studies reported that the sink rate of OLIF alone is about 32%–36.3%, higher than that of OLIF operation (29.9%) combined with internal fixation, but no significant correlation between fusion cage sink and clinical symptom improvement was found.^29^, ^30^ The risks of displacement include eccentric placement, uneven force in the absence of auxiliary internal fixation, and early activity, which lead to fusion cage displacement. Additionally, it is easy to form pseudarthrosis when the cage is not fused with the upper and lower vertebral bodies, resulting in displacement.^31^

### Several Considerations during OLIF Operation

OLIF approaches through intermuscular space and locates the diseased segment intervertebral space to treat intervertebral disc. It is generally believed that OLIF approaches through the natural space between retroperitoneum, psoas major muscle, and abdominal vessel, showing the advantages of minimal invasiveness, small incision, short operation time, less intraoperative blood loss, and quick postoperative recovery. Li *et al*.^15^ evaluated the development and application of OLIF and other traditional operations based on relevant recent literature, and they unveiled that OLIF has excellent orthopedic effects in ASD patients and the incidence of bony fusion is higher than for other approaches. Another meta‐analysis indicated the lengths of hospital stay and blood loss were better in OLIF than TLIF.^32^ Therefore, the patients can get down to the ground and do functional exercise early after OLIF. The self‐locking oblique lateral lumbar interbody fusion cage can achieve indirect decompression and relieve the symptoms of ASD without removing the previous internal fixation. According to the original intervertebral space height measured before the operation and the adjacent normal intervertebral space height, the degree of distraction was estimated to avoid over‐distraction of intervertebral space and reduce the risk of cage sink after the operation. During the preparation process, attention should be paid to protect the endplate. After scraping the cartilage endplate, the endplate should be made to seep blood slightly to facilitate bone ingrowth. Endplate injury is prone to cause fusion cage sink. Endplate injury includes iatrogenic injury, especially the endplate injury during the preparation of bone bed, and the endplate injury caused by osteoporosis and obesity. After preoperative and intraoperative assessment of the risk of cage sink, pedicle screw fixation can be used appropriately to provide more protection. The authors screened out two cases who received posterior internal fixation to avoid the cage sink because of endplate injury during the operation, and were not included in this study. The fusion cage should have its upper and lower parts fitted with the end plate and achieve optimization in the side and front and back coverage of the endplate, so as to improve the stability of the fusion cage and the success rate of fusion. Before the insertion of the inlays, fluoroscopy should be performed to confirm the appropriate position of the fusion cage to ensure that the fixation inlays can be implanted into the vertebral bodies. The inlays should be implanted according to the serial number on the holder to make the in lays completely implanted in the fusion cage. The inlays, fusion cage, and adjacent vertebral bodies can be anchored to each other.

### Different Operations in the Treatment of ASD after Lumbar Fusion

As one of the posterior surgical approaches for fusion, the TLIF approach is a direct and unilateral access to the intervertebral for aminal space. By removing the proliferated ligamentum flavum and hyperplastic facet process, TLIF can decompress the stenosis directly. OLIF uses the anatomical space between the aorta or inferior vena cava and the psoas muscle to access the disc space. Unlike the traditional posterior approaches, OLIF is less likely to damage the lamina, the paravertebral muscles, and the facet joints. TLIF leads to direct nerve decompression on the premise of partial resection of articular process and lamina, with more intuitive effects. Thereafter, the effects of different operation methods on the treatment of ASD after lumbar fusion were discussed. Louie *et al*.^33^ performed LLIF on 25 ASD patients after lumbar interbody fusion and suggested that LLIF and PLIF have the same clinical efficacy in the treatment of ASD after lumbar interbody fusion, but LLIF bears the advantages of shortening operation time and reducing operation complications. Zhu *et al*.^34^ compared the effects of OLIF and PLIF on ASD patients after the lumbar operation and found that OLIF is better than PLIF in improving perioperative parameters, promoting short‐term clinical efficacy, and restoring the height of intervertebral space. Wong *et al*.^35^ reported 11 cases of ASD treated with minimally invasive TLIF. Although the authors did not report the recovery of ASD patients in detail, MIS‐TLIF did show high safety, cause relatively less damage to paravertebral muscles, and exert exact decompression effect, representing a new therapeutic method for ASD after lumbar fusion. Nevertheless, no final conclusion has yet been reached on the efficacy of OLIF compared with TLIF in the treatment of ASD after lumbar fusion.

### Comparison of Clinical Efficacy between OLIF + Bridge‐Locking Cage and TLIF + Pedicle Screw Fixation

In this study, it was found that the intraoperative operation time, intraoperative blood loss, postoperative drainage, and hospital stay of patients in the OLIF group were shorter than those of patients in the TLIF group. The reasons for the increased bleeding in TLIF operation are as follows: the paravertebral muscles need to be separated during operation; some of scar tissues need to be resected; the upper articular process and part of the lower articular process and lamina need to be removed. The reasons for the prolonged operation time may be unclear vision field during the operation and the need to remove the previous internal fixation. However, there was no difference in VAS and ODI scores between the two groups of patients at different time points during follow‐up. The imaging results showed that the fusion rate of bone graft was 100% in both the OLIF group and TLIF group, and there was no difference in the fusion rate between the two groups of patients. As mentioned above, for the treatment of ASD, open operation again can increase the risk and difficulty of operation. Tormenti *et al*.^36^ evaluated 531 patients undergoing open posterior TLIF and found that the probability of perioperative complications in patients undergoing revision surgery was 1.75 times higher than that in patients undergoing primary surgery, and in particular, the risk of spinal dural tear caused by non‐iatrogenic injury is 1.75 times. Ogura *et al*.^37^ found that the average blood loss of the revision open posterior decompression and fusion surgery is 16% more than that of similar open lumbar fusion surgery. In this study, the postoperative complication rate of included patients who underwent TLIF was about 18%. Fortunately, 75% of these patients recovered with the aid of intensive exercise and nutritional nerve treatment. Notably, the rate of 18% is a little higher than the normal rate, which may be caused by the group of patients with the requirement of the second operation. In view of this, a comprehensive consideration should be made in the revision operation of ASD to achieve small injury, fast recovery, and good efficacy.

### Limitations and Strengths

There are some limitations in this study. First, the sample size of this study is relatively small and the follow‐up time is short, with the longest follow‐up time of 2 years. The long‐term efficacy needs further follow‐up analysis. As far as the current information, OLIF combined with the bridge‐locking cage has fewer complications. The adjacent segment degeneration caused by the bridge‐locking cage and related risk factors have not appeared yet. Also, there is not enough experience in the locking fusion revision. In future research, it is essential to pay attention to the issue of fusion cage sink in the medium and long term.

### Conclusion

To sum up, the innovation of this study lies in the comparison of the efficacy of OLIF and TLIF in the treatment of ASD after lumbar fusion. Our results manifested a significantly shorter operation time and length of stay in the OLIF patients than in TLIF. Besides, we also demonstrated that the intraoperative blood loss and postoperative drainage were less in OLIF patients, with a similar complication occurrence with TLIF. Therefore, the short‐term follow‐up efficacy of OLIF + bridge‐locking cage is satisfactory, which is worthy of promotion.

## Author Contributions

Shuai Zhang finished study design, Cheng‐hui Yin finished experimental studies, Xu Hui data analysis, Yin Cheng‐hui finished manuscript editing. All authors read and approved the final manuscript.
